# Supplementary material for: Effects of maternal healthcare service utilization on modern postpartum family planning access in Bangladesh: insights from a National representative survey
Source: PLoS One. 2025 Feb 4;20(2):e0318363. doi: 10.1371/journal.pone.0318363 (PMC12140116; doi:10.1371/journal.pone.0318363)
Supplement: S1 File — (DOCX) [file pone.0318363.s001.docx]

**Effects of Maternal Healthcare Service Utilization on Modern Postpartum Family Planning Access in Bangladesh: Insights from a National Representative Survey**


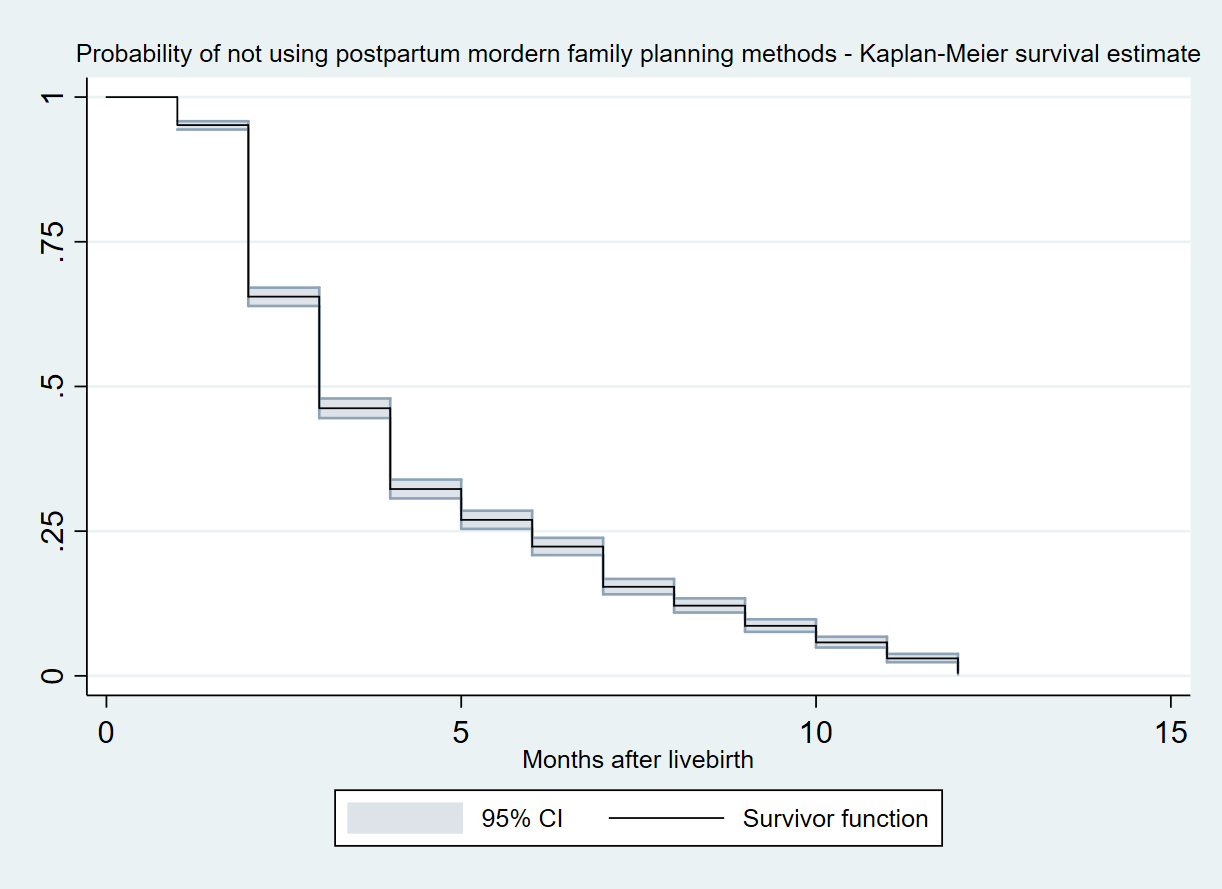


**Supplementary Figure 1.** Probability of not using postpartum modern family planning methods over the months after childbirth (1-12 months) using Kaplan Meier survival estimates

**Supplementary Table 1: Estimated VIF for multicollinearity for each of the exposure variable considered in the analysis**

|  | **At least 4 ANC with medically trained provider** | **Delivery by a skilled birth attendance** | **Delivery in healthcare facility** | **Caesarean delivery** | **Post-natal healthcare services** | **Continuity of care** |
| --- | --- | --- | --- | --- | --- | --- |
| Age at last child birth | 1.54 | 1.54 | 1.54 | 1.54 | 1.54 | 1.54 |
| Women’s education | 1.69 | 1.70 | 1.70 | 1.69 | 1.70 | 1.71 |
| Women’s working status | 1.10 | 1.10 | 1.10 | 1.10 | 1.09 | 1.09 |
| Parity | 1.64 | 1.64 | 1.64 | 1.64 | 1.64 | 1.64 |
| Women’s autonomy | 1.08 | 1.08 | 1.08 | 1.08 | 1.08 | 1.08 |
| Husband’s age | 1.65 | 1.65 | 1.65 | 1.65 | 1.65 | 1.65 |
| Husband’s education | 1.64 | 1.64 | 1.64 | 1.64 | 1.64 | 1.64 |
| Wealth quintile | 1.96 | 1.99 | 1.98 | 1.98 | 1.99 | 2.00 |
| Media exposure | 1.47 | 1.46 | 1.45 | 1.45 | 1.45 | 1.47 |
| Religion | 1.03 | 1.03 | 1.03 | 1.03 | 1.03 | 1.03 |
| Place of residence | 1.25 | 1.24 | 1.24 | 1.24 | 1.24 | 1.25 |
| Division | 1.03 | 1.03 | 1.03 | 1.03 | 1.03 | 1.03 |
| Terminated pregnancy | 1.04 | 1.04 | 1.04 | 1.04 | 1.04 | 1.04 |
| Menstrual resumption | 1.01 | 1.01 | 1.01 | 1.01 | 1.01 | 1.01 |
| Wanted last pregnancy |  |  | 1.06 |  |  |  |
| At least 4 ANC with medically trained provider | 1.21 |  |  |  |  |  |
| Delivery by a skilled birth attendance |  | 1.28 |  |  |  |  |
| Delivery in healthcare facility |  |  | 1.26 |  |  |  |
| Caesarean delivery |  |  |  | 1.22 |  |  |
| Post-natal healthcare services |  |  |  |  | 1.26 |  |
| Continuity of care |  |  |  |  |  | 1.38 |

**Supplementary Table 2:** Association of 4 ANC at least one with medically trained providers with postpartum modern family planning uptake among women who had given birth in three years preceding the survey using poison modified generalised linear model estimates: BDHS, 2017-18 (n=4081)

| **Variables** | **aPR** | **SE** | **z** | **P>z** | **95% Confidence Interval** | |
| --- | --- | --- | --- | --- | --- | --- |
|  |  |  |  |  | **Lower** | **Upper** |
| **Age at last child birth** |  |  |  |  |  |  |
| ≤19 years | 1.00 |  |  |  |  |  |
| 20-34 years | 0.96 | 0.03 | -1.35 | 0.176 | 0.91 | 1.02 |
| ≥35 years | 0.92 | 0.04 | -1.74 | 0.082 | 0.84 | 1.01 |
|  |  |  |  |  |  |  |
| **Women’s education** |  |  |  |  |  |  |
| No education | 1.00 |  |  |  |  |  |
| Primary | 0.99 | 0.03 | -0.28 | 0.779 | 0.94 | 1.05 |
| Secondary | 0.96 | 0.03 | -1.1 | 0.272 | 0.90 | 1.03 |
| Higher | 0.98 | 0.05 | -0.39 | 0.697 | 0.88 | 1.09 |
|  |  |  |  |  |  |  |
| **Women’s working status** |  |  |  |  |  |  |
| No | 1.00 |  |  |  |  |  |
| Yes | 0.99 | 0.02 | -0.31 | 0.757 | 0.96 | 1.03 |
|  |  |  |  |  |  |  |
| **Parity** |  |  |  |  |  |  |
| ≤2 | 1.00 |  |  |  |  |  |
| >2 | 1.03 | 0.03 | 0.92 | 0.359 | 0.97 | 1.08 |
|  |  |  |  |  |  |  |
| **Women’s autonomy** | **0.99** | **0.01** | **-2.35** | **0.019** | **0.97** | **1.00** |
|  |  |  |  |  |  |  |
| **Husband’s age** |  |  |  |  |  |  |
| <24 years | 1.00 |  |  |  |  |  |
| 25-34 years | 0.97 | 0.04 | -0.74 | 0.458 | 0.89 | 1.05 |
| 35-44 years | 0.92 | 0.04 | -2.07 | 0.038 | 0.84 | 1.00 |
| 45-59 years | 0.84 | 0.05 | -3.03 | 0.002 | 0.74 | 0.94 |
| 60+ years | 0.75 | 0.16 | -1.36 | 0.175 | 0.50 | 1.13 |
|  |  |  |  |  |  |  |
| **Husband’s education** |  |  |  |  |  |  |
| No education | 1.00 |  |  |  |  |  |
| Primary | 1.04 | 0.02 | 1.75 | 0.080 | 1.00 | 1.09 |
| Secondary | 1.00 | 0.03 | 0.15 | 0.885 | 0.95 | 1.06 |
| Higher | 1.00 | 0.03 | 0.03 | 0.973 | 0.95 | 1.05 |
| Don’t know | 0.68 | 0.14 | -1.9 | 0.057 | 0.46 | 1.01 |
|  |  |  |  |  |  |  |
| **Wealth quintile** |  |  |  |  |  |  |
| Poorest | 1.00 |  |  |  |  |  |
| Poorer | 0.95 | 0.04 | -1.05 | 0.294 | 0.87 | 1.04 |
| Middle | 0.91 | 0.03 | -2.82 | 0.005 | 0.86 | 0.97 |
| Richer | 0.90 | 0.02 | -4.05 | 0.000 | 0.85 | 0.95 |
| Richest | 0.91 | 0.04 | -2.14 | 0.032 | 0.84 | 0.99 |
|  |  |  |  |  |  |  |
| **Media exposure** |  |  |  |  |  |  |
| Low exposed | 1.00 |  |  |  |  |  |
| Moderately exposed | 1.07 | 0.02 | 3.21 | 0.001 | 1.03 | 1.12 |
| Highly exposed | 1.09 | 0.04 | 2.45 | 0.014 | 1.02 | 1.16 |
|  |  |  |  |  |  |  |
| **Religion** |  |  |  |  |  |  |
| Non-Muslim | 1.00 |  |  |  |  |  |
| Muslim | 1.13 | 0.06 | 2.27 | 0.023 | 1.02 | 1.25 |
|  |  |  |  |  |  |  |
| **Place of residence** |  |  |  |  |  |  |
| Urban | 1.00 |  |  |  |  |  |
| Rural | 0.93 | 0.02 | -4.22 | 0.000 | 0.90 | 0.96 |
|  |  |  |  |  |  |  |
| **Division** |  |  |  |  |  |  |
| Barishal | 1.00 |  |  |  |  |  |
| Chattogram | 0.91 | 0.02 | -4.93 | 0.000 | 0.87 | 0.94 |
| Dhaka | 0.97 | 0.01 | -2.39 | 0.017 | 0.95 | 1.00 |
| Khulna | 0.96 | 0.01 | -2.6 | 0.009 | 0.93 | 0.99 |
| Mymensingh | 1.02 | 0.01 | 2.88 | 0.004 | 1.01 | 1.04 |
| Rajshahi | 0.98 | 0.02 | -0.89 | 0.376 | 0.93 | 1.03 |
| Rangpur | 0.97 | 0.02 | -1.38 | 0.168 | 0.93 | 1.01 |
| Sylhet | 0.90 | 0.01 | -10.22 | 0.000 | 0.88 | 0.92 |
|  |  |  |  |  |  |  |
| **Terminated pregnancy** |  |  |  |  |  |  |
| No | 1.00 |  |  |  |  |  |
| Yes | 0.97 | 0.03 | -1.08 | 0.278 | 0.92 | 1.03 |
|  |  |  |  |  |  |  |
| **Menstrual resumption** |  |  |  |  |  |  |
| No | 1.00 |  |  |  |  |  |
| Yes | 1.45 | 0.08 | 6.45 | 0.000 | 1.30 | 1.62 |
|  |  |  |  |  |  |  |
| **Wanted last pregnancy** |  |  |  |  |  |  |
| Wanted later | 1.00 |  |  |  |  |  |
| Wanted then | 0.89 | 0.02 | -4.11 | 0.000 | 0.84 | 0.94 |
|  |  |  |  |  |  |  |
| **At least 4 ANC with medically trained provider** |  |  |  |  |  |  |
| No | 1.00 |  |  |  |  |  |
| Yes | 1.04 | 0.02 | 2.1 | 0.036 | 1.00 | 1.07 |
|  |  |  |  |  |  |  |
| Constant | 0.62 | 0.05 | -6.54 | 0.000 | 0.54 | 0.72 |
|  |  |  |  |  |  |  |
| **Model fitting estimates** |  |  |  |  |  |  |
| AIC | 7703.726 | | |  |  |  |
| BIC | 7798.204 | | |  |  |  |

**Supplementary Table 3:** Association of delivery by skilled birth attendant with postpartum modern family planning uptake among women who had given birth in three years preceding the survey using poison modified generalised linear model estimates: BDHS, 2017-18 (n=4081)

| **Variables** | **aPR** | | **SE** | **z** | **P>z** | | **95% Confidence Interval** | | | |
| --- | --- | --- | --- | --- | --- | --- | --- | --- | --- | --- |
|  |  | |  |  |  | | **Lower** | | **Upper** | |
| **Age at last child birth** |  | |  |  |  | |  | |  | |
| ≤19 years | 1.00 | |  |  |  | |  | |  | |
| 20-34 years | 0.96 | | 0.03 | -1.4 | 0.161 | | 0.91 | | 1.02 | |
| ≥35 years | 0.92 | | 0.04 | -1.81 | 0.070 | | 0.84 | | 1.01 | |
|  |  | |  |  |  | |  | |  | |
| **Women’s education** |  | |  |  |  | |  | |  | |
| No education | 1.00 | |  |  |  | |  | |  | |
| Primary | 0.99 | | 0.03 | -0.23 | 0.818 | | 0.94 | | 1.05 | |
| Secondary | 0.96 | | 0.03 | -1.15 | 0.250 | | 0.90 | | 1.03 | |
| Higher | 0.97 | | 0.05 | -0.51 | 0.611 | | 0.88 | | 1.08 | |
|  |  | |  |  |  | |  | |  | |
| **Women’s working status** |  | |  |  |  | |  | |  | |
| No | 1.00 | |  |  |  | |  | |  | |
| Yes | 1.00 | | 0.02 | -0.05 | 0.962 | | 0.96 | | 1.04 | |
|  |  | |  |  |  | |  | |  | |
| Parity |  | |  |  |  | |  | |  | |
| ≤2 |  | |  |  |  | |  | |  | |
| >2 | 1.03 | | 0.03 | 1.01 | 0.312 | | 0.97 | | 1.09 | |
|  |  | |  |  |  | |  | |  | |
| **Women’s autonomy** | **0.99** | | **0.01** | **-2.32** | **0.021** | | **0.97** | | **1.00** | |
|  |  | |  |  |  | |  | |  | |
| **Husband’s age** |  | |  |  |  | |  | |  | |
| <24 years | 1.00 | |  |  |  | |  | |  | |
| 25-34 years | 0.97 | | 0.04 | -0.74 | 0.460 | | 0.90 | | 1.05 | |
| 35-44 years | 0.92 | | 0.04 | -2.09 | 0.037 | | 0.84 | | 0.99 | |
| 45-59 years | 0.83 | | 0.05 | -3.11 | 0.002 | | 0.74 | | 0.93 | |
| 60+ years | 0.76 | | 0.16 | -1.32 | 0.187 | | 0.51 | | 1.14 | |
|  |  | |  |  |  | |  | |  | |
| **Husband’s education** |  | |  |  |  | |  | |  | |
| No education | 1.00 | |  |  |  | |  | |  | |
| Primary | 1.04 | | 0.02 | 1.76 | 0.079 | | 1.00 | | 1.09 | |
| Secondary | 1.00 | | 0.03 | 0.08 | 0.938 | | 0.95 | | 1.06 | |
| Higher | 1.00 | | 0.02 | -0.14 | 0.892 | | 0.95 | | 1.04 | |
| Don’t know | 0.68 | | 0.13 | -1.97 | 0.049 | | 0.46 | | 1.00 | |
|  |  | |  |  |  | |  | |  | |
| **Wealth quintile** |  | |  |  |  | |  | |  | |
| Poorest | 1.00 | |  |  |  | |  | |  | |
| Poorer | 0.95 | | 0.04 | -1.13 | 0.257 | | 0.87 | | 1.04 | |
| Middle | 0.91 | | 0.03 | -3.05 | 0.002 | | 0.85 | | 0.97 | |
| Richer | 0.89 | | 0.02 | -4.65 | 0.000 | | 0.85 | | 0.93 | |
| Richest | 0.90 | | 0.04 | -2.42 | 0.015 | | 0.83 | | 0.98 | |
|  |  | |  |  |  | |  | |  | |
| **Media exposure** |  | |  |  |  | |  | |  | |
| Low exposed | 1.00 | |  |  |  | |  | |  | |
| Moderately exposed | 1.07 | | 0.02 | 3.08 | 0.002 | | 1.02 | | 1.12 | |
| Highly exposed | 1.08 | | 0.04 | 2.32 | 0.021 | | 1.01 | | 1.16 | |
|  |  | |  |  |  | |  | |  | |
| **Religion** |  | |  |  |  | |  | |  | |
| Non-Muslim | 1.00 | |  |  |  | |  | |  | |
| Muslim | 1.13 | | 0.06 | 2.34 | 0.019 | | 1.02 | | 1.25 | |
|  |  | |  |  |  | |  | |  | |
| **Place of residence** |  | |  |  |  | |  | |  | |
| Urban | 1.00 | |  |  |  | |  | |  | |
| Rural | 0.93 | | 0.02 | -4.34 | 0.000 | | 0.90 | | 0.96 | |
|  |  | |  |  |  | |  | |  | |
| **Division** |  | |  |  |  | |  | |  | |
| Barishal | 1.00 | |  |  |  | |  | |  | |
| Chattogram | 0.91 | | 0.02 | -4.79 | 0.000 | | 0.87 | | 0.94 | |
| Dhaka | 0.98 | | 0.01 | -2.27 | 0.023 | | 0.96 | | 1.00 | |
| Khulna | 0.96 | | 0.02 | -2.41 | 0.016 | | 0.93 | | 0.99 | |
| Mymensingh | 1.03 | | 0.01 | 2.8 | 0.005 | | 1.01 | | 1.05 | |
| Rajshahi | 0.98 | | 0.02 | -0.86 | 0.392 | | 0.94 | | 1.03 | |
| Rangpur | 0.97 | | 0.02 | -1.14 | 0.256 | | 0.93 | | 1.02 | |
| Sylhet | 0.90 | | 0.01 | -9.61 | 0.000 | | 0.89 | | 0.92 | |
|  |  | |  |  |  | |  | |  | |
| **Terminated pregnancy** |  | |  |  |  | |  | |  | |
| No | 1.00 | |  |  |  | |  | |  | |
| Yes | 0.97 | | 0.03 | -1.2 | 0.229 | | 0.91 | | 1.02 | |
|  |  | |  |  |  | |  | |  | |
| **Menstrual resumption** |  | |  |  |  | |  | |  | |
| No | 1.00 | |  |  |  | |  | |  | |
| Yes | 1.46 | | 0.08 | 6.56 | 0.000 | | 1.30 | | 1.63 | |
|  |  | |  |  |  | |  | |  | |
| **Wanted last pregnancy** |  | |  |  |  | |  | |  | |
| Wanted later | 1.00 | |  |  |  | |  | |  | |
| Wanted then | 0.89 | | 0.03 | -4.08 | 0.000 | | 0.84 | | 0.94 | |
|  |  | |  |  |  | |  | |  | |
| **Delivery by a skilled birth attendance** |  | |  |  |  | |  | |  | |
| No | 1.00 | |  |  |  | |  | |  | |
| Yes | 1.07 | | 0.02 | 3.32 | 0.001 | | 1.03 | | 1.11 | |
|  |  | |  |  |  | |  | |  | |
| Constant | 0.61 | | 0.05 | -6.67 | 0.000 | | 0.53 | | 0.71 | |
|  |  | |  |  |  | |  | |  | |
| **Model fitting estimates** | |  | | | | | | | | |
| AIC | | 7702.146 | | | |  | |  | |  |
| BIC | | 7796.624 | | | |  | |  | |  |

**Supplementary Table 4:** Association of delivery in healthcare facility with postpartum modern family planning uptake among women who had given birth in three years preceding the survey using poison modified generalised linear model estimates: BDHS, 2017-18 (n=4081)

| **Variables** | **aPR** | | **SE** | **z** | **P>z** | **95% Confidence Interval** | | |  |
| --- | --- | --- | --- | --- | --- | --- | --- | --- | --- |
|  |  | |  |  |  | **Lower** | **Upper** | |  |
| **Age at last child birth** |  | |  |  |  |  |  | |  |
| ≤19 years | 1.00 | |  |  |  |  |  | |  |
| 20-34 years | 0.96 | | 0.03 | -1.4 | 0.161 | 0.91 | 1.02 | |  |
| ≥35 years | 0.92 | | 0.04 | -1.79 | 0.073 | 0.84 | 1.01 | |  |
|  |  | |  |  |  |  |  | |  |
| **Women’s education** |  | |  |  |  |  |  | |  |
| No education | 1.00 | |  |  |  |  |  | |  |
| Primary | 0.99 | | 0.03 | -0.22 | 0.822 | 0.94 | 1.05 | |  |
| Secondary | 0.96 | | 0.03 | -1.14 | 0.255 | 0.90 | 1.03 | |  |
| Higher | 0.97 | | 0.05 | -0.51 | 0.613 | 0.88 | 1.08 | |  |
|  |  | |  |  |  |  |  | |  |
| **Women’s working status** |  | |  |  |  |  |  | |  |
| No | 1.00 | |  |  |  |  |  | |  |
| Yes | 1.00 | | 0.02 | -0.02 | 0.983 | 0.96 | 1.04 | |  |
|  |  | |  |  |  |  |  | |  |
| **Parity** |  | |  |  |  |  |  | |  |
| ≤2 | 1.00 | |  |  |  |  |  | |  |
| >2 | 1.03 | | 0.03 | 1 | 0.316 | 0.97 | 1.09 | |  |
|  |  | |  |  |  |  |  | |  |
| **Women’s autonomy** | **0.99** | | **0.01** | **-2.3** | **0.021** | **0.97** | **1.00** | |  |
|  |  | |  |  |  |  |  | |  |
| **Husband’s age** |  | |  |  |  |  |  | |  |
| <24 years | 1.00 | |  |  |  |  |  | |  |
| 25-34 years | 0.97 | | 0.04 | -0.72 | 0.474 | 0.90 | 1.05 | |  |
| 35-44 years | 0.92 | | 0.04 | -2.07 | 0.039 | 0.84 | 1.00 | |  |
| 45-59 years | 0.83 | | 0.05 | -3.08 | 0.002 | 0.74 | 0.94 | |  |
| 60+ years | 0.76 | | 0.16 | -1.33 | 0.185 | 0.51 | 1.14 | |  |
|  |  | |  |  |  |  |  | |  |
| **Husband’s education** |  | |  |  |  |  |  | |  |
| No education | 1.00 | |  |  |  |  |  | |  |
| Primary | 1.04 | | 0.02 | 1.76 | 0.079 | 1.00 | 1.09 | |  |
| Secondary | 1.00 | | 0.03 | 0.08 | 0.932 | 0.95 | 1.06 | |  |
| Higher | 1.00 | | 0.02 | -0.1 | 0.920 | 0.95 | 1.05 | |  |
| Don’t know | 0.68 | | 0.13 | -1.98 | 0.048 | 0.46 | 1.00 | |  |
|  |  | |  |  |  |  |  | |  |
| **Wealth quintile** |  | |  |  |  |  |  | |  |
| Poorest | 1.00 | |  |  |  |  |  | |  |
| Poorer | 0.95 | | 0.04 | -1.11 | 0.267 | 0.87 | 1.04 | |  |
| Middle | 0.91 | | 0.03 | -2.98 | 0.003 | 0.85 | 0.97 | |  |
| Richer | 0.89 | | 0.02 | -4.55 | 0.000 | 0.85 | 0.94 | |  |
| Richest | 0.91 | | 0.04 | -2.35 | 0.019 | 0.84 | 0.98 | |  |
|  |  | |  |  |  |  |  | |  |
| **Media exposure** |  | |  |  |  |  |  | |  |
| Low exposed | 1.00 | |  |  |  |  |  | |  |
| Moderately exposed | 1.07 | | 0.02 | 3.11 | 0.002 | 1.03 | 1.12 | |  |
| Highly exposed | 1.09 | | 0.04 | 2.38 | 0.017 | 1.01 | 1.16 | |  |
|  |  | |  |  |  |  |  | |  |
| **Religion** |  | |  |  |  |  |  | |  |
| Non-Muslim | 1.00 | |  |  |  |  |  | |  |
| Muslim | 1.13 | | 0.06 | 2.35 | 0.019 | 1.02 | 1.25 | |  |
|  |  | |  |  |  |  |  | |  |
| **Place of residence** |  | |  |  |  |  |  | |  |
| Urban | 1.00 | |  |  |  |  |  | |  |
| Rural | 0.93 | | 0.02 | -4.33 | 0.000 | 0.90 | 0.96 | |  |
|  |  | |  |  |  |  |  | |  |
| **Division** |  | |  |  |  |  |  | |  |
| Barishal | 1.00 | |  |  |  |  |  | |  |
| Chattogram | 0.91 | | 0.02 | -4.89 | 0.000 | 0.87 | 0.94 | |  |
| Dhaka | 0.97 | | 0.01 | -2.63 | 0.009 | 0.95 | 0.99 | |  |
| Khulna | 0.96 | | 0.02 | -2.55 | 0.011 | 0.93 | 0.99 | |  |
| Mymensingh | 1.02 | | 0.01 | 2.56 | 0.010 | 1.01 | 1.04 | |  |
| Rajshahi | 0.98 | | 0.02 | -1.02 | 0.307 | 0.93 | 1.02 | |  |
| Rangpur | 0.97 | | 0.02 | -1.32 | 0.187 | 0.93 | 1.01 | |  |
| Sylhet | 0.90 | | 0.01 | -10.09 | 0.000 | 0.88 | 0.92 | |  |
|  |  | |  |  |  |  |  | |  |
| **Terminated pregnancy** |  | |  |  |  |  |  | |  |
| No | 1.00 | |  |  |  |  |  | |  |
| Yes | 0.97 | | 0.03 | -1.23 | 0.218 | 0.91 | 1.02 | |  |
|  |  | |  |  |  |  |  | |  |
| **Menstrual resumption** |  | |  |  |  |  |  | |  |
| No | 1.00 | |  |  |  |  |  | |  |
| Yes | 1.46 | | 0.08 | 6.53 | 0.000 | 1.30 | 1.63 | |  |
|  |  | |  |  |  |  |  | |  |
| **Wanted last pregnancy** |  | |  |  |  |  |  | |  |
| Wanted later | 1.00 | |  |  |  |  |  | |  |
| Wanted then | 0.89 | | 0.03 | -4.07 | 0.000 | 0.84 | 0.94 | |  |
|  |  | |  |  |  |  |  | |  |
| **Delivery in healthcare facility** |  | |  |  |  |  |  | |  |
| No | 1.00 | |  |  |  |  |  | |  |
| Yes | 1.06 | | 0.02 | 3.09 | 0.002 | 1.02 | 1.10 | |  |
|  |  | |  |  |  |  |  | |  |
| Constant | 0.61 | | 0.05 | -6.65 | 0.000 | 0.53 | 0.71 | |  |
|  |  | |  |  |  |  |  | |  |
| **Model fitting estimates** | |  | | | | | | | |
| AIC | | 7702.381 | | |  |  | |  | |
| BIC | | 7796.859 | | |  |  | |  | |

**Supplementary Table 5:** Association of caesarean delivery with postpartum modern family planning uptake among women who had given birth in three years preceding the survey using poison modified generalised linear model estimates: BDHS, 2017-18 (n=4081)

| **Variables** | **aPR** | | **SE** | **z** | **P>z** | | **[95% Confidence Interval]** | | | | |
| --- | --- | --- | --- | --- | --- | --- | --- | --- | --- | --- | --- |
|  |  | |  |  |  | | **Lower** | | **Upper** | | |
| **Age at last child birth** |  | |  |  |  | |  | |  | | |
| ≤19 years | 1.00 | |  |  |  | |  | |  | | |
| 20-34 years | 0.96 | | 0.03 | -1.45 | 0.148 | | 0.91 | | 1.02 | | |
| ≥35 years | 0.92 | | 0.04 | -1.82 | 0.069 | | 0.83 | | 1.01 | | |
|  |  | |  |  |  | |  | |  | | |
| **Women’s education** |  | |  |  |  | |  | |  | | |
| No education | 1.00 | |  |  |  | |  | |  | | |
| Primary | 1.00 | | 0.03 | -0.17 | 0.867 | | 0.95 | | 1.05 | | |
| Secondary | 0.96 | | 0.03 | -1.05 | 0.295 | | 0.90 | | 1.03 | | |
| Higher | 0.97 | | 0.05 | -0.48 | 0.635 | | 0.88 | | 1.08 | | |
|  |  | |  |  |  | |  | |  | | |
| **Women’s working status** |  | |  |  |  | |  | |  | | |
| No | 1.00 | |  |  |  | |  | |  | | |
| Yes | 1.00 | | 0.02 | -0.07 | 0.945 | | 0.96 | | 1.04 | | |
|  |  | |  |  |  | |  | |  | | |
| **Parity** |  | |  |  |  | |  | |  | | |
| ≤2 | 1.00 | |  |  |  | |  | |  | | |
| >2 | 1.03 | | 0.03 | 0.99 | 0.321 | | 0.97 | | 1.09 | | |
|  |  | |  |  |  | |  | |  | | |
| **Women’s autonomy** | **0.99** | | **0.01** | **-2.23** | **0.026** | | **0.97** | | **1.00** | | |
|  |  | |  |  |  | |  | |  | | |
| **Husband’s age** |  | |  |  |  | |  | |  | | |
| <24 years | 1.00 | |  |  |  | |  | |  | | |
| 25-34 years | 0.97 | | 0.04 | -0.79 | 0.431 | | 0.89 | | 1.05 | | |
| 35-44 years | 0.91 | | 0.04 | -2.14 | 0.033 | | 0.84 | | 0.99 | | |
| 45-59 years | 0.83 | | 0.05 | -3.11 | 0.002 | | 0.74 | | 0.93 | | |
| 60+ years | 0.76 | | 0.16 | -1.34 | 0.180 | | 0.51 | | 1.14 | | |
|  |  | |  |  |  | |  | |  | | |
| **Husband’s education** |  | |  |  |  | |  | |  | | |
| No education | 1.00 | |  |  |  | |  | |  | | |
| Primary | 1.04 | | 0.02 | 1.75 | 0.081 | | 1.00 | | 1.09 | | |
| Secondary | 1.00 | | 0.03 | 0.09 | 0.927 | | 0.95 | | 1.06 | | |
| Higher | 0.99 | | 0.02 | -0.28 | 0.781 | | 0.95 | | 1.04 | | |
| Don’t know | 0.68 | | 0.13 | -2.03 | 0.043 | | 0.46 | | 0.99 | | |
|  |  | |  |  |  | |  | |  | | |
| **Wealth quintile** |  | |  |  |  | |  | |  | | |
| Poorest | 1.00 | |  |  |  | |  | |  | | |
| Poorer | 0.95 | | 0.04 | -1.09 | 0.278 | | 0.87 | | 1.04 | | |
| Middle | 0.91 | | 0.03 | -3.02 | 0.003 | | 0.85 | | 0.97 | | |
| Richer | 0.89 | | 0.02 | -4.4 | 0.000 | | 0.85 | | 0.94 | | |
| Richest | 0.90 | | 0.04 | -2.49 | 0.013 | | 0.83 | | 0.98 | | |
|  |  | |  |  |  | |  | |  | | |
| **Media exposure** |  | |  |  |  | |  | |  | | |
| Low exposed | 1.00 | |  |  |  | |  | |  | | |
| Moderately exposed | 1.07 | | 0.02 | 2.96 | 0.003 | | 1.02 | | 1.12 | | |
| Highly exposed | 1.09 | | 0.04 | 2.27 | 0.023 | | 1.01 | | 1.16 | | |
|  |  | |  |  |  | |  | |  | | |
| **Religion** |  | |  |  |  | |  | |  | | |
| Non-Muslim | 1.00 | |  |  |  | |  | |  | | |
| Muslim | 1.13 | | 0.06 | 2.36 | 0.019 | | 1.02 | | 1.25 | | |
|  |  | |  |  |  | |  | |  | | |
| **Place of residence** |  | |  |  |  | |  | |  | | |
| Urban | 1.00 | |  |  |  | |  | |  | | |
| Rural | 0.93 | | 0.02 | -4.46 | 0.000 | | 0.90 | | 0.96 | | |
|  |  | |  |  |  | |  | |  | | |
| **Division** |  | |  |  |  | |  | |  | | |
| Barishal | 1.00 | |  |  |  | |  | |  | | |
| Chattogram | 0.91 | | 0.02 | -4.8 | 0.000 | | 0.87 | | 0.94 | | |
| Dhaka | 0.97 | | 0.01 | -2.66 | 0.008 | | 0.95 | | 0.99 | | |
| Khulna | 0.96 | | 0.02 | -2.64 | 0.008 | | 0.93 | | 0.99 | | |
| Mymensingh | 1.02 | | 0.01 | 2.5 | 0.012 | | 1.00 | | 1.04 | | |
| Rajshahi | 0.98 | | 0.02 | -1.01 | 0.311 | | 0.93 | | 1.02 | | |
| Rangpur | 0.97 | | 0.02 | -1.14 | 0.253 | | 0.93 | | 1.02 | | |
| Sylhet | 0.90 | | 0.01 | -9.63 | 0.000 | | 0.88 | | 0.92 | | |
|  |  | |  |  |  | |  | |  | | |
| **Terminated pregnancy** |  | |  |  |  | |  | |  | | |
| No | 1.00 | |  |  |  | |  | |  | | |
| Yes | 0.97 | | 0.03 | -1.21 | 0.225 | | 0.91 | | 1.02 | | |
|  |  | |  |  |  | |  | |  | | |
| **Menstrual resumption** |  | |  |  |  | |  | |  | | |
| No | 1.00 | |  |  |  | |  | |  | | |
| Yes | 1.46 | | 0.09 | 6.42 | 0.000 | | 1.30 | | 1.63 | | |
|  |  | |  |  |  | |  | |  | | |
| **Wanted last pregnancy** |  | |  |  |  | |  | |  | | |
| Wanted later | 1.00 | |  |  |  | |  | |  | | |
| Wanted then | 0.89 | | 0.03 | -4.15 | 0.000 | | 0.84 | | 0.94 | | |
|  |  | |  |  |  | |  | |  | | |
| **Caesarean delivery** |  | |  |  |  | |  | |  | | |
| No | 1.00 | |  |  |  | |  | |  | | |
| Yes | 1.08 | | 0.02 | 3.57 | 0.000 | | 1.03 | | 1.12 | | |
|  |  | |  |  |  | |  | |  | | |
| Constant | 0.62 | | 0.05 | -6.58 | 0.000 | | 0.54 | | 0.72 | | |
|  |  | |  |  |  | |  | |  | | |
| **Model fitting estimates** | |  | | | | | | | | |  |
| AIC | | 7693.009 | | | |  | |  | |  |  |
| BIC | | 7787.472 | | | |  | |  | |  |  |

**Supplementary Table 6:** Association of postnatal healthcare services with postpartum modern family planning uptake among women who had given birth in three years preceding the survey using poison modified generalised linear model estimates: BDHS, 2017-18 (n=4081)

| **Variables** | **aPR** | | **SE** | **z** | **P>z** | | **95% Confidence Interval** | | | | |
| --- | --- | --- | --- | --- | --- | --- | --- | --- | --- | --- | --- |
|  |  | |  |  |  | | **Lower** | | **Upper** | | |
| **Age at last child birth** |  | |  |  |  | |  | |  | | |
| ≤19 years | 1.00 | |  |  |  | |  | |  | | |
| 20-34 years | 0.96 | | 0.03 | -1.4 | 0.163 | | 0.91 | | 1.02 | | |
| ≥35 years | 0.92 | | 0.04 | -1.79 | 0.074 | | 0.84 | | 1.01 | | |
|  |  | |  |  |  | |  | |  | | |
| **Women’s education** |  | |  |  |  | |  | |  | | |
| No education | 1.00 | |  |  |  | |  | |  | | |
| Primary | 0.99 | | 0.03 | -0.22 | 0.829 | | 0.94 | | 1.05 | | |
| Secondary | 0.96 | | 0.03 | -1.13 | 0.260 | | 0.90 | | 1.03 | | |
| Higher | 0.97 | | 0.05 | -0.48 | 0.628 | | 0.88 | | 1.08 | | |
|  |  | |  |  |  | |  | |  | | |
| **Women’s working status** |  | |  |  |  | |  | |  | | |
| No | 1.00 | |  |  |  | |  | |  | | |
| Yes | 1.00 | | 0.02 | -0.07 | 0.942 | | 0.96 | | 1.04 | | |
|  |  | |  |  |  | |  | |  | | |
| **Parity** |  | |  |  |  | |  | |  | | |
| ≤2 | 1.00 | |  |  |  | |  | |  | | |
| >2 | 1.03 | | 0.03 | 0.99 | 0.322 | | 0.97 | | 1.09 | | |
|  |  | |  |  |  | |  | |  | | |
| **Women’s autonomy** | **0.99** | | **0.01** | **-2.31** | **0.021** | | **0.97** | | **1.00** | | |
|  |  | |  |  |  | |  | |  | | |
| **Husband’s age** |  | |  |  |  | |  | |  | | |
| <24 years | 1.00 | |  |  |  | |  | |  | | |
| 25-34 years | 0.97 | | 0.04 | -0.73 | 0.468 | | 0.90 | | 1.05 | | |
| 35-44 years | 0.92 | | 0.04 | -2.08 | 0.037 | | 0.84 | | 0.99 | | |
| 45-59 years | 0.83 | | 0.05 | -3.13 | 0.002 | | 0.74 | | 0.93 | | |
| 60+ years | 0.76 | | 0.16 | -1.32 | 0.186 | | 0.51 | | 1.14 | | |
|  |  | |  |  |  | |  | |  | | |
| **Husband’s education** |  | |  |  |  | |  | |  | | |
| No education | 1.00 | |  |  |  | |  | |  | | |
| Primary | 1.04 | | 0.02 | 1.76 | 0.079 | | 1.00 | | 1.09 | | |
| Secondary | 1.00 | | 0.03 | 0.07 | 0.945 | | 0.95 | | 1.06 | | |
| Higher | 1.00 | | 0.02 | -0.12 | 0.907 | | 0.95 | | 1.05 | | |
| Don’t know | 0.68 | | 0.13 | -1.97 | 0.049 | | 0.46 | | 1.00 | | |
|  |  | |  |  |  | |  | |  | | |
| **Wealth quintile** |  | |  |  |  | |  | |  | | |
| Poorest | 1.00 | |  |  |  | |  | |  | | |
| Poorer | 0.95 | | 0.04 | -1.12 | 0.261 | | 0.87 | | 1.04 | | |
| Middle | 0.91 | | 0.03 | -3.06 | 0.002 | | 0.85 | | 0.97 | | |
| Richer | 0.89 | | 0.02 | -4.6 | 0.000 | | 0.85 | | 0.94 | | |
| Richest | 0.90 | | 0.04 | -2.44 | 0.015 | | 0.83 | | 0.98 | | |
|  |  | |  |  |  | |  | |  | | |
| **Media exposure** |  | |  |  |  | |  | |  | | |
| Low exposed | 1.00 | |  |  |  | |  | |  | | |
| Moderately exposed | 1.07 | | 0.02 | 3.14 | 0.002 | | 1.03 | | 1.12 | | |
| Highly exposed | 1.09 | | 0.04 | 2.39 | 0.017 | | 1.02 | | 1.16 | | |
|  |  | |  |  |  | |  | |  | | |
| **Religion** |  | |  |  |  | |  | |  | | |
| Non-Muslim | 1.00 | |  |  |  | |  | |  | | |
| Muslim | 1.13 | | 0.06 | 2.33 | 0.020 | | 1.02 | | 1.25 | | |
|  |  | |  |  |  | |  | |  | | |
| **Place of residence** |  | |  |  |  | |  | |  | | |
| Urban | 1.00 | |  |  |  | |  | |  | | |
| Rural | 0.93 | | 0.02 | -4.34 | 0.000 | | 0.90 | | 0.96 | | |
|  |  | |  |  |  | |  | |  | | |
| **Division** |  | |  |  |  | |  | |  | | |
| Barishal | 1.00 | |  |  |  | |  | |  | | |
| Chattogram | 0.91 | | 0.02 | -4.78 | 0.000 | | 0.87 | | 0.94 | | |
| Dhaka | 0.98 | | 0.01 | -2.24 | 0.025 | | 0.96 | | 1.00 | | |
| Khulna | 0.96 | | 0.02 | -2.47 | 0.014 | | 0.93 | | 0.99 | | |
| Mymensingh | 1.03 | | 0.01 | 2.79 | 0.005 | | 1.01 | | 1.05 | | |
| Rajshahi | 0.98 | | 0.02 | -0.9 | 0.369 | | 0.94 | | 1.03 | | |
| Rangpur | 0.97 | | 0.02 | -1.17 | 0.242 | | 0.93 | | 1.02 | | |
| Sylhet | 0.90 | | 0.01 | -9.69 | 0.000 | | 0.89 | | 0.92 | | |
|  |  | |  |  |  | |  | |  | | |
| **Terminated pregnancy** |  | |  |  |  | |  | |  | | |
| No | 1.00 | |  |  |  | |  | |  | | |
| Yes | 0.97 | | 0.03 | -1.21 | 0.227 | | 0.91 | | 1.02 | | |
|  |  | |  |  |  | |  | |  | | |
| **Menstrual resumption** |  | |  |  |  | |  | |  | | |
| No | 1.00 | |  |  |  | |  | |  | | |
| Yes | 1.46 | | 0.08 | 6.54 | 0.000 | | 1.30 | | 1.63 | | |
|  |  | |  |  |  | |  | |  | | |
| **Wanted last pregnancy** |  | |  |  |  | |  | |  | | |
| Wanted later | 1.00 | |  |  |  | |  | |  | | |
| Wanted then | 0.89 | | 0.03 | -4.1 | 0.000 | | 0.84 | | 0.94 | | |
|  |  | |  |  |  | |  | |  | | |
| **Post-natal healthcare services** |  | |  |  |  | |  | |  | | |
| No | 1.00 | |  |  |  | |  | |  | | |
| Yes | 1.06 | | 0.02 | 3.63 | 0.000 | | 1.03 | | 1.10 | | |
|  |  | |  |  |  | |  | |  | | |
| Constant | 0.61 | | 0.05 | -6.67 | 0.000 | | 0.53 | | 0.71 | | |
|  |  | |  |  |  | |  | |  | | |
| **Model fitting estimates** | |  | | | | | | | | |  |
| AIC | | 7702.308 | | | |  | |  | |  |  |
| BIC | | 7796.786 | | | |  | |  | |  |  |

**Supplementary Table 7:** Association of continuity of care with postpartum modern family planning uptake among women who had given birth in three years preceding the survey using poison modified generalised linear model estimates: BDHS, 2017-18 (n=4081)

| **Variables** | **aPR** | | **SE** | **z** | **P>z** | | **95% Confidence Interval** | | | | |
| --- | --- | --- | --- | --- | --- | --- | --- | --- | --- | --- | --- |
|  |  | |  |  |  | | **Lower** | | | **Upper** | |
| **Age at last child birth** |  | |  |  |  | |  | | |  | |
| ≤19 years | 1.00 | |  |  |  | |  | | |  | |
| 20-34 years | 0.96 | | 0.03 | -1.39 | 0.165 | | 0.91 | | | 1.02 | |
| ≥35 years | 0.92 | | 0.04 | -1.77 | 0.076 | | 0.84 | | | 1.01 | |
|  |  | |  |  |  | |  | | |  | |
| **Women’s education** |  | |  |  |  | |  | | |  | |
| No education | 1.00 | |  |  |  | |  | | |  | |
| Primary | 0.99 | | 0.03 | -0.31 | 0.753 | | 0.94 | | | 1.04 | |
| Secondary | 0.96 | | 0.03 | -1.25 | 0.211 | | 0.90 | | | 1.02 | |
| Higher | 0.97 | | 0.05 | -0.56 | 0.575 | | 0.88 | | | 1.08 | |
|  |  | |  |  |  | |  | | |  | |
| **Women’s working status** |  | |  |  |  | |  | | |  | |
| No | 1.00 | |  |  |  | |  | | |  | |
| Yes | 1.00 | | 0.02 | -0.19 | 0.849 | | 0.96 | | | 1.03 | |
|  |  | |  |  |  | |  | | |  | |
| **Parity** |  | |  |  |  | |  | | |  | |
| ≤2 | 1.00 | |  |  |  | |  | | |  | |
| >2 | 1.03 | | 0.03 | 0.99 | 0.325 | | 0.97 | | | 1.09 | |
|  |  | |  |  |  | |  | | |  | |
| **Women’s autonomy** | **0.99** | | **0.01** | **-2.29** | **0.022** | | **0.97** | | | **1.00** | |
|  |  | |  |  |  | |  | | |  | |
| **Husband’s age** |  | |  |  |  | |  | | |  | |
| <24 years | 1.00 | |  |  |  | |  | | |  | |
| 25-34 years | 0.97 | | 0.04 | -0.66 | 0.510 | | 0.89 | | | 1.06 | |
| 35-44 years | 0.92 | | 0.04 | -2.03 | 0.042 | | 0.84 | | | 1.00 | |
| 45-59 years | 0.83 | | 0.05 | -3.02 | 0.003 | | 0.74 | | | 0.94 | |
| 60+ years | 0.76 | | 0.16 | -1.33 | 0.183 | | 0.50 | | | 1.14 | |
|  |  | |  |  |  | |  | | |  | |
| **Husband’s education** |  | |  |  |  | |  | | |  | |
| No education | 1.00 | |  |  |  | |  | | |  | |
| Primary | 1.04 | | 0.02 | 1.74 | 0.081 | | 1.00 | | | 1.09 | |
| Secondary | 1.00 | | 0.03 | 0 | 0.996 | | 0.95 | | | 1.05 | |
| Higher | 0.99 | | 0.02 | -0.29 | 0.769 | | 0.95 | | | 1.04 | |
| Don’t know | 0.68 | | 0.14 | -1.94 | 0.053 | | 0.46 | | | 1.00 | |
|  |  | |  |  |  | |  | | |  | |
| **Wealth quintile** |  | |  |  |  | |  | | |  | |
| Poorest | 1.00 | |  |  |  | |  | | |  | |
| Poorer | 0.95 | | 0.04 | -1.09 | 0.277 | | 0.87 | | | 1.04 | |
| Middle | 0.91 | | 0.03 | -2.99 | 0.003 | | 0.85 | | | 0.97 | |
| Richer | 0.89 | | 0.02 | -4.49 | 0.000 | | 0.85 | | | 0.94 | |
| Richest | 0.90 | | 0.04 | -2.51 | 0.012 | | 0.83 | | | 0.98 | |
|  |  | |  |  |  | |  | | |  | |
| **Media exposure** |  | |  |  |  | |  | | |  | |
| Low exposed | 1.00 | |  |  |  | |  | | |  | |
| Moderately exposed | 1.07 | | 0.02 | 3.09 | 0.002 | | 1.02 | | | 1.12 | |
| Highly exposed | 1.08 | | 0.04 | 2.26 | 0.024 | | 1.01 | | | 1.15 | |
|  |  | |  |  |  | |  | | |  | |
| **Religion** |  | |  |  |  | |  | | |  | |
| Non-Muslim | 1.00 | |  |  |  | |  | | |  | |
| Muslim | 1.13 | | 0.06 | 2.34 | 0.019 | | 1.02 | | | 1.25 | |
|  |  | |  |  |  | |  | | |  | |
| **Place of residence** |  | |  |  |  | |  | | |  | |
| Urban | 1.00 | |  |  |  | |  | | |  | |
| Rural | 0.93 | | 0.02 | -4.19 | 0.000 | | 0.90 | | | 0.96 | |
|  |  | |  |  |  | |  | | |  | |
| **Division** |  | |  |  |  | |  | | |  | |
| Barishal | 1.00 | |  |  |  | |  | | |  | |
| Chattogram | 0.91 | | 0.02 | -4.86 | 0.000 | | 0.88 | | | 0.94 | |
| Dhaka | 0.98 | | 0.01 | -2.24 | 0.025 | | 0.96 | | | 1.00 | |
| Khulna | 0.96 | | 0.02 | -2.5 | 0.012 | | 0.93 | | | 0.99 | |
| Mymensingh | 1.02 | | 0.01 | 2.61 | 0.009 | | 1.01 | | | 1.04 | |
| Rajshahi | 0.98 | | 0.02 | -0.94 | 0.348 | | 0.93 | | | 1.02 | |
| Rangpur | 0.97 | | 0.02 | -1.4 | 0.160 | | 0.93 | | | 1.01 | |
| Sylhet | 0.90 | | 0.01 | -9.48 | 0.000 | | 0.89 | | | 0.92 | |
|  |  | |  |  |  | |  | | |  | |
| **Terminated pregnancy** |  | |  |  |  | |  | | |  | |
| No | 1.00 | |  |  |  | |  | | |  | |
| Yes | 0.97 | | 0.03 | -1.2 | 0.229 | | 0.91 | | | 1.02 | |
|  |  | |  |  |  | |  | | |  | |
| **Menstrual resumption** |  | |  |  |  | |  | | |  | |
| No | 1.00 | |  |  |  | |  | | |  | |
| Yes | 1.45 | | 0.08 | 6.47 | 0.000 | | 1.30 | | | 1.63 | |
|  |  | |  |  |  | |  | | |  | |
| **Wanted last pregnancy** |  | |  |  |  | |  | | |  | |
| Wanted later | 1.00 | |  |  |  | |  | | |  | |
| Wanted then | 0.89 | | 0.03 | -4.17 | 0.000 | | 0.84 | | | 0.94 | |
|  |  | |  |  |  | |  | | |  | |
| **Continuity of care** |  | |  |  |  | |  | | |  | |
| Low (ref) | 1.00 | |  |  |  | |  | | |  | |
| Moderate | 1.02 | | 0.03 | 0.56 | 0.574 | | 0.96 | | | 1.08 | |
| High | 1.09 | | 0.02 | 4.41 | 0.000 | | 1.05 | | | 1.13 | |
|  |  | |  |  |  | |  | | |  | |
| Constant | 0.62 | | 0.04 | -6.89 | 0.000 | | 0.54 | | | 0.71 | |
|  |  | |  |  |  | |  | | |  | |
| **Model fitting estimates** | |  | | | | | | | | |  |
| AIC | | 7701.742 | | | |  | |  |  | |  |
| BIC | | 7796.220 | | | |  | |  |  | |  |
