# Supplementary material for: Effects of maternal healthcare service utilization on modern postpartum family planning access in Bangladesh: insights from a National representative survey
Source: PLoS One. 2025 Feb 4;20(2):e0318363. doi: 10.1371/journal.pone.0318363 (PMC12140116; doi:10.1371/journal.pone.0318363)
Supplement: Attachment — Submitted filename: Response to reviwers.docx [file pone.0318363.s002.docx]

**Reviewers’ comments and author’s responses**

| **Reviewers’ comments** | **Authors’ response** |
| --- | --- |
| **Reviewer#1** |  |
| Did the assumptions of the model fit? Effect modification? Multi-collinearity? | We assessed the model assumptions and evaluated multicollinearity before initiating each model. The following sentences are included in the statistical analysis section to provide clarity:  *We used the Poisson modified generalised linear regression model with clustered error variance because it produces a more precise result compared to logistic regression analysis as the survey we analysed had a hierarchical structure and a prevalence of maternal healthcare services use was over 10% [29]. We ran both unadjusted and adjusted models. In the unadjusted model, each form of maternal healthcare services use was considered with the outcome variable without any confounding variables. In the adjusted model, confounding variables were considered. Before executing the models, we conducted a multicollinearity check utilizing Variance Inflation Factors (VIF). If we detected evidence of multicollinearity, specifically if VIF exceeded 10, we proceeded to remove the pertinent variables and then re-ran the models. All analyses accounted for the complex survey design and sampling weights.* |
| Provide residual plots of the assumption. | We appreciate your feedback regarding the presentation of residual plots for the multiple Poisson regression model. In this specific context, we have chosen not to include residual plots for several reasons. Firstly, residual plots are traditionally used in linear regression and may not be directly applicable to multiple Poisson regression, as the assumptions and diagnostic criteria differ. In the case of Poisson regression, other diagnostic measures such as goodness-of-fit tests, deviance statistics, and overdispersion assessments are more relevant and informative. Secondly, the concept of homoscedasticity, which residual plots are designed to assess in linear regression, is not as applicable to Poisson regression. Poisson models inherently account for heteroscedasticity by assuming that the variance is proportional to the mean. Additionally, dichotomized data, which we analyzed in this study using a Poisson regression model, can be highly skewed or contain numerous zero counts. In such cases, residual plots may not effectively capture the characteristics of the data. Instead, we have used other appropriate diagnostic tools and assessments to ensure the validity and robustness of our multiple Poisson regression analysis. |
| If the variables were not significant in the unadjusted model, why then adjusted in the multiple model? | We did not include any variables that were found to be statistically insignificant in the adjusted models. We have made revisions to the section detailing the adjusted variables to ensure clarity. The newly added sentences are as follows:  *We conducted a three-stage process to identify the variables for adjustment. First, we conducted an extensive literature search for relevant studies in LMICs and Bangladesh [5, 14, 15, 17, 18, 20, 21, 26-28]. Based on this literature review, we generated a list of potential adjustment variables, which were subsequently verified for their presence in the survey dataset. The variables that were found to be available in the survey were then assessed for their statistical significance in relation to the outcome variables. Finally, only those variables that exhibited statistical significance in forward regression were considered for inclusion in the analysis.*  We also acknowledge that reviewers may raise concerns, particularly regarding the outcome variable "4 ANC, at least one with medically trained providers," which showed an association that was not statistically significant, although it came very close to the significance level. However, it's essential to emphasize that this is our chosen outcome variable. In statistical analysis, it is not appropriate to dismiss an adjusted association solely because it lacks strong statistical significance. It's worth noting that even considering a 0.20 significance level as a threshold for variable selection in adjusted models is a common practice in epidemiological research and is statistically valid. |
| In multivariable regression, variable selection requires justification like check of confounding, interaction, and association in bivariate regression along with the support of the literature. | We have provided justification on variable selection in both the adjusted variables and statistical analysis section. In the adjusted variables section, we have the following sentences:  *We conducted a three-stage process to identify the variables for adjustment. First, we conducted an extensive literature search for relevant studies in LMICs and Bangladesh [5, 14, 15, 17, 18, 20, 21, 26-28]. Based on this literature review, we generated a list of potential adjustment variables, which were subsequently verified for their presence in the survey dataset. The variables that were found to be available in the survey were then assessed for their statistical significance in relation to the outcome variables. Finally, only those variables that exhibited statistical significance in forward regression were considered for inclusion in the analysis….*  In the statistical analysis section, we have now the following sentences:  *…….. We ran both unadjusted and adjusted models. In the unadjusted model, each form of maternal healthcare services use was considered with the outcome variable without any confounding variables. In the adjusted model, confounding variables were considered. Before executing the models, we conducted a multicollinearity check utilizing Variance Inflation Factors (VIF). If we detected evidence of multicollinearity, specifically if VIF exceeded 10, we proceeded to remove the pertinent variables and then re-ran the models. All analyses accounted for the complex survey design and sampling weights.………….* |
| After fitting the model, did author check the model fitness? Report AIC, BIC. | We evaluated the model fitness after running each model, and they demonstrated a good fit. Nevertheless, we did not initially report metrics such as AIC and BIC, as these are typically not included in the type of study we conducted. However, in response to your comment, we have now included AIC and BIC for all models. Please see the revised version of the supplementary tables. |
| Post estimation test, VIF require to do to check for the collinearity. | We conducted checks for multicollinearity before running each model, and if evidence of multicollinearity was detected, we removed the relevant variables and reran the models. To address this matter, we have included the following sentences in the statistical analysis section of the revised version of the manuscript.  *Before executing the models, we conducted a multicollinearity check utilizing Variance Inflation Factors (VIF). If we detected evidence of multicollinearity, specifically if VIF exceeded 10, we proceeded to remove the pertinent variables and then re-ran the models.* |
| The ROC curve with the AUC should be reported to make sure that the models predicted the outcome. | ROC curves are primarily employed to evaluate the performance of classification models, such as logistic regression models. In the context of this study, Poisson regression models were used, which are better suited for analysing count data and rate-based outcomes. Additionally, the assessment and reporting of AUC (area under the curve), which measures a model's discriminatory power, are not applicable to this study. Therefore, we have not made any specific changes to address this comment. |
| "As such, the ‘event’ (failure) was postpartum modern contraceptive uptake, and the ‘duration’ (time) was measured up to the first 12 months (follow-up period) following the most recent live birth" in BDHS event and time are measured at the same time (single interview), so, the duration is not possible to measure as the temporality is not exist (it is ecological). so, the attempt of using KM may not have the luxury of validity. The author needs to address this and provide a transparent explanation. | We would like to emphasize to the reviewer that in this study, we analyzed calendar data. This approach involves collecting monthly data on reproductive history, extending five years back from the survey time. For each month within this period, respondents reported data on contraception use or non-use, including the type of contraception used. More information on this approach can be found in the DHS content available at http://dhsprogram.com/pubs/pdf/CR36/CR36.pdf.  As a result, your observation regarding the validity of KM is not applicable to our study. However, in response to your comment, we have included the following sentences in the outcome variable section of the revised manuscript to ensure that this point is clearly articulated within the document.  ………… *To generate this variable, we used women’s reproductive calendar data, taking into account the month they started using FP methods following a live birth and the types of FP methods used. In this particular form of data collection technique, women were asked to document their monthly history of contraception use or non-use and provide reasons for non-use or discontinuation. A list of contraceptive methods available in Bangladesh at the time of the survey, as outlined in Table 2, was provided to assist reporting. Respondents were also given the option to indicate if the contraceptive methods they used were not listed. We reclassified these responses into the utilization of modern FP methods (including the pill, IUD, injectables, condoms, female sterilization, male sterilization, and implant, coded as 1), and other methods (coded as 0), adhering to the WHO's categorization guidelines. We also extracted data on the duration (in months) of staring modern FP use, from the date of the most recent childbirth within the first 12 months postpartum period with the possibility of censoring.* |
| Reviewer#2 |  |
| Title is original and specific but not time bound. Results discussion and conclusion are based on data generated and well written | Thank you for your time to review our paper. |
| Tables need to be submitted separately | According to PLOS One's submission guidelines, tables must be submitted with the main manuscript. Therefore, we have not made any changes. |
| Overall it is good research in short of minor problems which could be easily edited | We have thoroughly rechecked the entire manuscript, and we have addressed the identified issues. |
| Reviewer#3 |  |
| Title: Your title clear and self-explanatory | No specific change is required to address this comment. |
| Abstract -The abstract is well written  -Avoid abbreviation and acronyms from abstract | We have removed all abbreviations and acronyms from the abstract. |
| Introduction -Well written, except some grammar error | We have conducted a thorough review of the entire manuscript and have addressed any grammatical errors that were found. |
| Methods |  |
| The methodology part is well stated with some concerns | We have addressed your concerns and provided a detailed point-by-point response below. The relevant changes have been incorporated into the revised version of the manuscript. |
| - the way your screened the study participants were clear and self-explanatory | Thank you. We have made further edits to enhance clarity and improve readability. |
| - You classified your outcome variable as used modern family planning during post-partum period, even the post-partum period you defined was highly extended. The contraceptive methods you classified as modern family planning were short acting (POP, COC, Depo-Provera) Long acting (Implants (Norplant) and sterilization (female and male sterilization). | The timeframe we considered for the postpartum period aligns with the recommendations of the World Health Organization. With regards to the classification of contraception, we followed the practice of grouping both short-acting and long-acting contraceptive methods together, categorizing them as modern contraception methods. This categorization is in accordance with the WHO's guidance on contraception classification. It is worth noting that classifying them as short-acting or long-acting contraception methods could have been an alternative approach. However, due to the limited representation of long-acting contraception methods in our dataset, approximately 5% of the total sample, conducting the analysis using this sub-sample was not feasible. We have edited the outcome variable section to ensure clarity in the procedure for variable creation. The revised section now reads as follows:  *The primary outcome variable for this study was modern PPFP methods use during the postpartum period of 0-12 months post-delivery as recommended by the WHO [23]. To generate this variable, we used women’s reproductive calendar data, taking into account the month they started using FP methods following a live birth and the types of FP methods used. In this particular form of data collection technique, women were asked to document their monthly history of contraception use or non-use and provide reasons for non-use or discontinuation. A list of contraceptive methods available in Bangladesh at the time of the survey, as outlined in Table 2, was provided to assist reporting. Respondents were also given the option to indicate if the contraceptive methods they used were not listed. We reclassified these responses into the utilization of modern FP methods (including the pill, IUD, injectables, condoms, female sterilization, male sterilization, and implant, coded as 1), and other methods (coded as 0), adhering to the WHO's categorization guidelines. We also extracted data on the duration (in months) of staring modern FP use, from the date of the most recent childbirth within the first 12 months postpartum period with the possibility of censoring.* |
| How you see the Norplant methods? Currently, WHO do not recommend norplant contraceptive in steady, there are safe and effective as Norplant are available like Jeddele, Sinoplant and implanon). Please clearly state if there are no other implants were not available in Bangladesh during this study period, unless your source of data is under question. | Thank you for your valuable input. The survey gathered data on Norplant, and the contraceptive methods you mentioned were not accessible in Bangladesh at the time of the survey. |
| What about Lactational Amenorrhea Method(LAM)? | Only two respondents from our initially selected sample for analysis reported Lactational Amenorrhea Method (LAM) use, and they also met our other exclusion criteria outlined in the study participation section. As a result, these two respondents were automatically excluded from the analysis. |
| Are you considered LAM method? | Only two respondents from our initially selected sample for analysis reported Lactational Amenorrhea Method (LAM) use, and they also met our other exclusion criteria outlined in the study participation section. As a result, these two respondents were automatically excluded from the analysis. |
| Exposure variables |  |
| Utilization of maternal healthcare services during pregnancy, child birth and post-partum period are obviously known as it increases post-partum utilization of family planning. No need to prove by research. Better if you saw its effectiveness. | Thank you for your insights. In this study, as you recommended, we examined the effect of maternal healthcare services on postpartum family planning uptake. We have taken care to make this aspect readily comprehensible across the entire manuscript during our editing process. |
| It is too vague to see all maternal health service as one exposure variable. | We have examined various forms of maternal healthcare services as seperate outcome variables. However, the variable you referred to, where all forms of maternal healthcare services are considered together, is known as "continuity of care." This aspect has garnered significant attention in the global health arena due to its effectiveness in reducing adverse maternal and child health outcomes. Both the World Health Organization and the Sustainable Development Goals recommend the importance of continuity of care, making it a significant focus. Therefore, we are introducing this concept here, as we believe it has substantial policy implications. Consequently, we have decided not to make specific changes to address this comment. |
| When we see ANC has four visits according to your study, currently, WHO recommended eight contacts. In your study, number of visit not considered at what visit post family planning is focused? | We acknowledge that the World Health Organization (WHO) now recommends eight ANC visits. However, it's important to note that in Bangladesh, the government's official recommendation remains at four ANC visits. A very small percentage of women in Bangladesh meet or exceed this eight-visit threshold, and this group is predominantly composed of individuals with higher education and socio-economic status. Given this context, we have opted to retain our previous classification and have not made any specific changes.  We already have the following sentence to present it into the manuscript-  *The WHO’s guideline 2014 stated that during the course of a pregnancy, each woman should: i) receive at least four skilled ANC visits (this was revised to eight ANC visits in 2016, however, Bangladesh’s government still follow the recommendation of four visits)* |
| The very important exposure component of maternal health service is post-partum care specially fourth visit of post-partum care. Why you considered post-partum visit within two days mean first visit? Other components of maternal health service have on impact on post-partum contraceptive use.  Why you categorized mode of delivery as a component of maternal health service (Delivery at health facility and Caesarian section)? | We recognize the special importance of the fourth postpartum care visit in relation to postpartum contraception uptake. In our analysis, we initially planned to incorporate it. However, it's essential to note that the utilization of postnatal care is very infrequent in Bangladesh, and as such, the survey did not collect detailed data on postnatal care visits. Instead, the survey focused solely on whether respondents received any postnatal care and the timing of the first check (i.e., the day after delivery). In line with the World Health Organization's (WHO) recommendations for postnatal care, we reclassified these responses into those who received postnatal care within two days and those who did not. Given this data limitation, we were unable to make changes to address the first part of your comment. However, we have acknowledged this limitation in the revised version of the manuscript by adding the following sentences to the strengths and limitations section:  *………….Furthermore, the global literature has demonstrated the effectiveness of four postnatal care visits in increasing the adoption of modern FP methods. Unfortunately, we were unable to consider this factor due to a lack of available data……………..*  As for the second part regarding the mode of delivery, it's worth noting that healthcare facility deliveries in Bangladesh are often the result of caesarean sections. Even in the case of normal deliveries, women typically receive postnatal care before leaving hospitals. Therefore, healthcare facilities in Bangladesh have policies in place to discuss family planning and contraception with women before their discharge. We took this into consideration in our analysis. |
| Adjusted variable- How you classify women’s working status as Yes/ No response? | The classification was derived from the survey data. In the survey, women were queried about their involvement in formal income-generating occupations, with responses recorded as either "yes" or "no." |
| Analysis methods and models you used is interested – Poison regression model is appropriate for this study. | Thank you. |
| Result |  |
| Result part is well written including tables and figures. | No specific change is required to address this comment. |
| Discussion |  |
| -lacks comparing with the existing data/ literatures | There is a scarcity of relevant research in LMICs and Bangladesh. We have considered and compared only a few studies that are available. However, we have conducted a thorough re-evaluation and ensured that all available data are presented in the revised version of the manuscript. Please see the revised version of the manuscript. |
| -lacks justifications | We have edited the relevant section and provide enough justification. Please see the discussion section of the revised version of the manuscript. |
| -Needs clearly stating the implication of your finding | We have now added a separate policy implication section in the discussion section of the revised manuscript to address this comment. This reads-  *The findings of this study suggest important policy implications for increasing the uptake of PPFP. It is evident that the current approach of increasing PPFP through access to maternal healthcare services is not as effective as desired; their role is relatively minor but still significant. Therefore, there is an urgent need to address the challenges within maternal healthcare sector to enhance counselling on PPFP. This includes increasing the coverage of maternal healthcare services and the number of healthcare personnel dedicated to provide maternal healthcare. Furthermore, PPFP counselling should be integrated as a vital component of the services provided by private healthcare facilities in Bangladesh. Challenges associated with providing and ensuring maternal healthcare services and FP in government healthcare facilities also need to be addressed. This involves ensuring proper integration between the DGHS and the DGFP and addressing structural issues, such as designating specific areas for PPFP services* |
| Conclusion |  |
| Please conclude your finding with the existing context | We have included the following sentence in the conclusion section of the revised manuscript to address this comment:  *Enhancing maternal healthcare services, integrating PPFP as a component of services offered by private healthcare facilities, and addressing structural challenges are crucial steps to improve the uptake PPFP.* |
| # Please, revise all the documents regarding grammar and spelling error | We have conducted a thorough review of the entire manuscript and have addressed any grammatical errors that were found. |
