# Supplementary material for: Effects of maternal healthcare service utilization on modern postpartum family planning access in Bangladesh: insights from a National representative survey
Source: PLoS One. 2025 Feb 4;20(2):e0318363. doi: 10.1371/journal.pone.0318363 (PMC12140116; doi:10.1371/journal.pone.0318363)
Supplement: Attachment — Submitted filename: manuscript review.docx [file pone.0318363.s003.docx]

**Title:** **Maternal Healthcare Service Utilization and Modern Postpartum Family Planning Discussion**

****Abstract:****

The study aims to investigate the association between maternal healthcare service utilization and the adoption of modern family planning (FP) methods in the postpartum period in Bangladesh. While the prevalence of postpartum FP uptake was reported at 73%, the dominant methods were pills and condoms. The results suggest that maternal healthcare service utilization has only a modest effect on increasing the uptake of modern FP methods during the postpartum period (3%-7%). The study highlights significant challenges, including limited counseling on modern FP methods during maternal healthcare services, a delay in the initiation of FP methods post-birth, and a concerning reliance on less effective contraceptive methods. The findings have implications for achieving Bangladesh's national and SDG goals related to maternal and child health.

**Strengths:**

1. **Large and Representative Sample:** The study analyzed a large nationally representative sample, enhancing the generalizability of the findings.

2. **Adjustment for Confounding Variables:** The results are adjusted for a wide range of confounding variables at the individual, household, and community levels through advanced statistical modeling.

3. **Adherence to WHO Recommendations:** The study adhered to WHO recommendations in generating variables for modern FP methods and maternal healthcare service use.

**Limitations:**

1. **Cross-Sectional Nature:** The cross-sectional design limits the ability to establish causal relationships between maternal healthcare service utilization and FP method uptake.

2. **Retrospective Data Collection:** Both maternal healthcare services and contraception uptake data were collected retrospectively, introducing potential recall bias.

3. **Incomplete Consideration of Factors:** Community-level norms and traditions, and healthcare facility availability and readiness, were not fully considered due to a lack of relevant data.

4. **Exclusion of Postnatal Care Visits:** The study did not consider the effectiveness of postnatal care visits, a factor shown to influence the adoption of modern FP methods globally.

5. **type of study, place of study, and number of participants should be mentioned in the abstract

**Recommendations:**

1. **Enhanced Counseling:** Urgent measures are needed to address the challenges within the maternal healthcare sector, emphasizing the importance of counseling on postpartum family planning.

2. **Integration of Private Healthcare Facilities:** The study suggests integrating postpartum family planning counseling as a vital component of services provided by private healthcare facilities.

3. **Addressing Structural Challenges:** Efforts should be made to improve coordination between the Directorate General of Family Planning (DGFP) and the Directorate General of Health Services (DGHS), addressing structural challenges and ensuring appropriate designated areas for postpartum family planning services.

**Review and Critique:**

**Introduction:**

The introduction provides a comprehensive overview of the background, emphasizing the importance of reducing maternal and child mortality in low- and middle-income countries (LMICs) and the role of family planning (FP) in achieving this goal. It effectively highlights the persisting challenges of unintended pregnancies and short birth intervals in LMICs, particularly during the extended postpartum period. The link between the non-use of FP methods and adverse outcomes is well-established. The section also contextualizes the situation in Bangladesh, stating its high rates of maternal and child mortality, unintended pregnancies, and short birth intervals.

**Strengths of the Introduction:**

1. **Clear Rationale:** The introduction effectively justifies the study by emphasizing the urgent need for modern postpartum family planning (PPFP) in LMICs, with a specific focus on Bangladesh.

2. **Relevance to SDG 3:** The connection between the study and Sustainable Development Goal 3 is well-established, demonstrating the alignment of the research with global health priorities.

**Critique of the Introduction:**

1. **Data Sources:** While the introduction mentions that the study analyzed data from the 2017-18 Bangladesh Demographic Health Survey (BDHS), it would be beneficial to briefly describe the survey methodology to enhance transparency.

2. **Updates on Global Guidelines:** Since the study refers to WHO guidelines, a brief mention of any updates or changes to these guidelines after 2018 would add currency to the context.

**Methodology:**

The methodology section provides a detailed account of the study design, data source, study participants, outcome and exposure variables, adjusted variables, and statistical analysis. The use of the 2017-18 BDHS data is appropriate for a nationally representative study. The criteria for participant selection are clearly outlined, and the inclusion and exclusion process is well-defined. The statistical methods, including Kaplan-Meier survival curves and modified Poisson regression, are appropriate for analyzing time-to-event data and handling the complex survey structure.

**Strengths of the Methodology:**

1. **Transparent Selection Criteria:** The criteria for selecting study participants are clear, contributing to the study's internal validity.

2. **Appropriate Statistical Methods:** The use of Kaplan-Meier survival curves and Poisson regression for analyzing time-to-event data is suitable for the research objectives.

**Critique of the Methodology:**

1. **Multicollinearity Check:** While the methodology mentions a multicollinearity check using Variance Inflation Factors (VIF), it would be helpful to report the results of this check or mention that no significant multicollinearity was found.

2. **Clarity on Duration:** The description of the "duration" in the time-to-event analysis could be further clarified for readers unfamiliar with this statistical approach.

Overall, the manuscript provides a comprehensive overview of the study, and the introduction and methodology are well-structured. Below are some feedback and suggestions on the Results and Discussion sections:

\### Results:

1. **Clarity in Presentation:**

- The background characteristics are well-presented in Table 1. However, consider breaking it down into sub-sections to improve readability. For example, you could have sub-sections for demographic characteristics and maternal healthcare utilization.

2. **Figures and Tables:**

- Figures 2, 3, 4, and 5 are referred to but not provided in the document. Make sure to include these figures for a complete understanding of the results.

- Ensure that the captions for figures and tables are sufficiently detailed to make them self-explanatory.

3. **Survival Probability Analysis:**

- The interpretation of the survival probability results could be made clearer. Explain the implications of the probabilities at different time points more explicitly.

\### Discussion:

1. **Structure:**

- Consider breaking down the discussion into sub-sections to address specific aspects, such as challenges in maternal healthcare services, individual-level factors, and policy implications. This will enhance the organization and readability of the discussion.

2. **Linking to Literature:**

- Strengthen the discussion by linking your findings to existing literature more explicitly. If your results align or contrast with previous studies, discuss these points in detail.

3. **Policy Implications:**

- While you discuss policy implications, consider providing more actionable recommendations. How can policymakers address the identified challenges, and what specific changes could be made based on your findings?

4. **Addressing Limitations:**

- You've appropriately discussed the limitations of your study. It might be useful to discuss potential avenues for future research that could address these limitations.

5. **Causality and Temporality:**

- Acknowledge the cross-sectional nature of your study and the limitations it imposes on establishing causality. Discuss the need for prospective studies to confirm the observed associations.

6. **Language and Clarity:**

- Some sentences are complex and might benefit from simplification. Ensure that each point is expressed clearly and concisely.

7. **Conclusion:**

- The conclusion could be more concise and reiterate the key findings and their policy implications.

Remember that these are suggestions, and you should adapt them according to the specific guidelines of the journal you are submitting to. Overall, your manuscript is well-structured and addresses an important public health issue. Good luck with your submission!
