# Supplementary material for: Effects of maternal healthcare service utilization on modern postpartum family planning access in Bangladesh: insights from a National representative survey
Source: PLoS One. 2025 Feb 4;20(2):e0318363. doi: 10.1371/journal.pone.0318363 (PMC12140116; doi:10.1371/journal.pone.0318363)
Supplement: Attachment — Submitted filename: Response_to_reviwers_auresp_2.docx [file pone.0318363.s005.docx]

**Reviewers’ comments and author’s responses**

| **Reviewers’ comments** | **Authors’ response** |
| --- | --- |
| **Reviewer#5** |  |
| Your title should be modified in line with your objective, dependent variable and study protocol | Modern postpartum family planning access was the dependent variable of this study, whereas maternal healthcare services utilization was the independent variable. Both are mentioned in the title. However, we have revised the title as follows to make it clearer in line with your comment:  *Effects of Maternal Healthcare Service Utilization on Modern Postpartum Family Planning Access in Bangladesh: Insights from a National Representative Survey* |
| Your background part; result and conclusion part mainly talks to family planning only where other maternal health care service is because it is huge.  Start your background part with statement of maternal health care service introduction then burden and its prevalence global to local context, strategies to overcome problems…. Flow | Thank you for this observation. The focus of this paper is postpartum family planning services uptake. As per the PLoS ONE publication guidelines, like other scientific journals, in the background section, authors are to provide broad details about the focus areas, i.e., modern postpartum family planning services access. This includes current conditions from a global to local context. Then the journal suggests how explanatory variables are linked with such scenarios, i.e., strategies to improve. We followed this guideline, which was also somewhat matched with your recommendation. In the background section, the first three paragraphs focus on postpartum modern family planning access, its current conditions globally and locally, and how it impacts maternal and child health. Then, in the fourth paragraph, we linked postpartum family planning access with maternal healthcare services use.  According to the journal guidelines, we are not allowed to mainly focus on maternal healthcare services use. Therefore, we did not make any changes in this section. However, we agree that your suggested area is important and deserves further study. |
| In your study participant inclusion criteria what about unmarried but uses FP and maternal service | In Bangladesh, sex outside marriage is completely restricted, and any evidence of it remains completely unreported. Moreover, the survey we analyzed collected data only from married women. We clearly mentioned this in the data source section of the methods by adding the following sentence:  *………. The inclusion criteria for completion of this survey were: (i) ever-married women aged 15-49 years and (ii) staying in the selected households on the night preceding the survey.…………..*  We therefore did not make any change to address this comment. |
| What is your study objective? | The study objective mentioned at the end of the background section of the manuscript. It reads-  *………………….We therefore aimed to determine the effects of maternal healthcare services use, including antenatal care (ANC), delivery with skilled birth attendants (SBA), delivery care in healthcare facilities (DHC), caesarean section delivery and postnatal care (PNC), on modern PPFP methods uptake in Bangladesh…………………………..* |
| What is your dependent variable? | By the nature of this study and the factors we analyzed, using the term "dependent variable" is not allowed. Instead, the term "outcome variable" is more applicable. As such, we used this term, which is similar to the term "dependent variable" you asked for. Please see the outcome variable section of the methods. |
| What was your study design | This is a cross-sectional study. We have now edited relevant sentence in the data source section of the methods section to make it clear. This now reads-  *This study analysed national representative cross-sectional survey data from the 2017-18 Bangladesh Demographic Health Survey (BDHS), conducted as part of the Demographic and Health Survey (DHS) program.* |
| What was your study period? | Study period is mentioned in the first sentences of the data source section of the methods section. They read-  *This study analysed national representative cross-sectional survey data from the 2017-18 Bangladesh Demographic Health Survey (BDHS), conducted as part of the Demographic and Health Survey (DHS) program. Data collection took place between September to December 2017.* |
| Is your study homogenous? | We analysed nationally representative survey data. This is not homogeneous. |
| Please rewrite your result part to show clearly what socio-demographic part, obstetric and gynecological history…. | We did not consider obstetrical and gynaecological history in our model. Furthermore, these data were not available in the survey. However, the socio-demographic factors we considered were only for adjustment purposes, and focusing on them would alter the study focus. Therefore, we did not emphasize them, though we discuss them where necessary. There is already ample literature from Bangladesh and LMICs covering socio-demographic determinants of postpartum modern contraception uptake, and interested readers can refer to them. We have cited them throughout our manuscript. |
| Your discussion, conclusion and recommendation should be in line with your finding only | We have checked our discussion, conclusion and recommendation sections and ensured they are line of the study findings. Thanks for your comment. |
| Ethical consideration needed to use such like data | We already included the ethical approval and consent to participation section at the end of the manuscript, where we mentioned that ethical approval was obtained by the survey authority. However, we extracted data from the survey authority in de-identified form. As we found the data to be in de-identified form, obtaining another ethical approval is not required, as it aligns with international practices regarding ethical issues. We have now clarified this issue in the manuscript by editing the aforementioned section. This now reads:  **Ethics approval and consent to participate:** The data of this study were obtained from MEASURE DHS Archive, and originally collected by the Macro, Calverton, USA. ORC Macro Institutional Review Board reviewed and approved the data collection procedure. Informed consent was obtained from each participant prior to enrolment. We obtained de-identified data by submitting research proposal to MEASURE DHS to conducted this study. No further ethical approval was therefore required. |
| Put brief description of study area | This is a nationally representative study for Bangladesh, and as such, the entire country is the study area. We have already provided a comprehensive description regarding Bangladesh in the introduction section and the data source section. Additionally, some information is also presented in the discussion section. Please have a look. |
| Reviewer#6 |  |
| Surely, I would be pleased to assess this work that is being considered for publication in Plosone.  To editor I think this paper is also presented in “the lancet library” https://papers.ssrn.com/sol3/papers.cfm?abstract_id=4463501 and check for grammatical issue (I could not access word doc.) | es, the paper is available on the Lancet server as a pre-print. According to the publication guidelines of PLoS ONE, authors are allowed and always encouraged to publish pre-print versions.  We have thoroughly checked the manuscript and corrected the grammatical errors. |
| **Comments to Author(s)**  **General comments** |  |
| Dear author, I tried to find what by mean “modern family planning” through your document please operationalize it  2. There is no line number for your document to comment it by line | We have sentences in the outcome variable section of the methods to clarify it. We have copied the most relevant sentences and pasted here for your quick look-    *Respondents were also given the option to indicate if the contraceptive methods they used were not listed. We reclassified these responses according to WHO's categorization guidelines into the utilization of modern FP methods (which included the pill, intra-uterine devices (IUDs), injectables, condoms, female sterilization, male sterilization, and implant, coded as 1) and other methods (coded as 0).*  Please see the aforementioned section for more details. Although adding line numbers is not required by PLoS ONE, we have included them in the revised version of the manuscript. |
| **Abstract** |  |
| 1. Your background does not give any clues towards effects of using modern family planning or lack of it◊modify it | We have edited the background section of the manuscript as follows to address your comment:  *Access to modern family planning is critical for improving maternal and child health outcomes, yet it remains severely lacking in low- and middle-income countries, including Bangladesh. Maternal healthcare utilization during and after pregnancy is vital for promoting postpartum family planning. This study examined the effects of maternal healthcare service utilization on postpartum family planning uptake in Bangladesh.* |
| 2. Your way of reporting “result” is good◊add contributing/associated factors here with its appropriate statistics | Thanks. No change is required to address this comment. |
| 3. Put your keywords in alphabetical order | We have rearranged the keywords alphabetical order as you recommended. |
| **Methods** |  |
| 1. Dear Author, you believe that information gathered six years ago can be used to draw conclusions that policymakers could use it | Thank you for bringing up this important point. Although the data was collected six years earlier, it was released in 2022, and since then, it represents the most recent data available in Bangladesh. The current round of BDHS is still undergoing, and it will require a significant amount of time before its release. Therefore, we do not have any alternative but to consider this data. |
| 2. What is your criteria to categorize media exposure to “no, moderate & highly exposed” (has no sense)??? | We have now edited the relevant sentences to make it clear. The relevant edited sentence now reads-  *…………………* *Husband’s age at the time of survey (<24 years, 25-34 years, 35-44 years, ≥45 years), husband’s education (no education, primary, secondary, higher), parity (≤2, >2), media exposure (not exposed (i.e., reported no access to radio/television/newspapers within a week of the survey), moderately exposed (i.e., reported at least one day but less than 3 days access to radio/television/newspapers within a week of the survey), and highly exposed (if respondents reported regular access to radio/television/newspapers within a week of the survey)) and pregnancy intention (wanted, unwanted) were household level characteristics included in the model. ……………………..* |
| **Discussion** |  |
| 1. Result & discussion well written◊ in discussion you did not discuss about contributing factors ----why???? | Thank you for this important observation. We did not give more focus to the contributing factors directly because they were not the aim of this study. Instead, we focused more on the issue of modern family planning, which was the aim of this study, and linked it with contributing factors. Numerous studies have been published in Bangladesh and other LMICs focusing on the socio-demographic determinants of modern family planning services uptake, which we adjusted in the model. We have cited these studies throughout the manuscript. Interested readers can learn more about them from these published papers. Focusing on them would make our study similar to them. |
| 2. Conclusion ◊ so what should will happen or for whom/what is your recommendation | We have edited relevant sentences in the conclusion section to make them clearer. The edited version of the sentence now reads:  *Enhancing maternal healthcare services, integrating PPFP as a component of services offered by private healthcare facilities, and addressing structural challenges are recommended to improve the uptake PPFP. This however requires a greater focus on the healthcare service delivery system.* |
| **Abstract:**  The study aims to investigate the association between maternal healthcare service utilization and the adoption of modern family planning (FP) methods in the postpartum period in Bangladesh. While the prevalence of postpartum FP uptake was reported at 73%, the dominant methods were pills and condoms. The results suggest that maternal healthcare service utilization has only a modest effect on increasing the uptake of modern FP methods during the postpartum period (3%-7%). The study highlights significant challenges, including limited counseling on modern FP methods during maternal healthcare services, a delay in the initiation of FP methods post-birth, and a concerning reliance on less effective contraceptive methods. The findings have implications for achieving Bangladesh's national and SDG goals related to maternal and child health.  **Strengths:**  1. **Large and Representative Sample:** The study analyzed a large nationally representative sample, enhancing the generalizability of the findings.  2. **Adjustment for Confounding Variables:** The results are adjusted for a wide range of confounding variables at the individual, household, and community levels through advanced statistical modeling.  3. **Adherence to WHO Recommendations:** The study adhered to WHO recommendations in generating variables for modern FP methods and maternal healthcare service use.  **Limitations:**  1. **Cross-Sectional Nature:** The cross-sectional design limits the ability to establish causal relationships between maternal healthcare service utilization and FP method uptake.  2. **Retrospective Data Collection:** Both maternal healthcare services and contraception uptake data were collected retrospectively, introducing potential recall bias.  3. **Incomplete Consideration of Factors:** Community-level norms and traditions, and healthcare facility availability and readiness, were not fully considered due to a lack of relevant data.  4. **Exclusion of Postnatal Care Visits:** The study did not consider the effectiveness of postnatal care visits, a factor shown to influence the adoption of modern FP methods globally.  **5. **type of study, place of study, and number of participants should be mentioned in the abstract**  **Recommendations:**  1. **Enhanced Counseling:** Urgent measures are needed to address the challenges within the maternal healthcare sector, emphasizing the importance of counseling on postpartum family planning.  2. **Integration of Private Healthcare Facilities:** The study suggests integrating postpartum family planning counseling as a vital component of services provided by private healthcare facilities.  3. **Addressing Structural Challenges:** Efforts should be made to improve coordination between the Directorate General of Family Planning (DGFP) and the Directorate General of Health Services (DGHS), addressing structural challenges and ensuring appropriate designated areas for postpartum family planning services. | No change is required to address these general observations.  We have now ensured these information are available in the revised version of the manuscript. Please have a look. |
| **Introduction:**  The introduction provides a comprehensive overview of the background, emphasizing the importance of reducing maternal and child mortality in low- and middle-income countries (LMICs) and the role of family planning (FP) in achieving this goal. It effectively highlights the persisting challenges of unintended pregnancies and short birth intervals in LMICs, particularly during the extended postpartum period. The link between the non-use of FP methods and adverse outcomes is well-established. The section also contextualizes the situation in Bangladesh, stating its high rates of maternal and child mortality, unintended pregnancies, and short birth intervals.  **Strengths of the Introduction:**  1. **Clear Rationale:** The introduction effectively justifies the study by emphasizing the urgent need for modern postpartum family planning (PPFP) in LMICs, with a specific focus on Bangladesh.  2. **Relevance to SDG 3:** The connection between the study and Sustainable Development Goal 3 is well-established, demonstrating the alignment of the research with global health priorities.  **Critique of the Introduction:**   1. **Data Sources:** While the introduction mentions that the study analyzed data from the 2017-18 Bangladesh Demographic Health Survey (BDHS), it would be beneficial to briefly describe the survey methodology to enhance transparency.   2. **Updates on Global Guidelines:** Since the study refers to WHO guidelines, a brief mention of any updates or changes to these guidelines after 2018 would add currency to the context. | The section we mentioned already provides a comprehensive overview of the survey methodology. Please refer to lines 89-114 in the revised version of the manuscript.  We already mentioned the most recent recommendation that WHO have so far. We have copy-pasted the relevant sentence here from the exposure variable section of the methods-  *The WHO’s 2014 guideline stated that during the course of a pregnancy, each woman should: i) receive at least four skilled ANC visits (while this was revised to eight ANC visits in 2016, Bangladesh’s government still follow the recommendation of four visits); ii) be assisted by a SBA during delivery; and iii) receive at least one PNC visit within 2 days of delivery from skilled healthcare personnel.*  Please see lines 155 to 167 in the revised version of the manuscript for details. |
| **Methodology:**  The methodology section provides a detailed account of the study design, data source, study participants, outcome and exposure variables, adjusted variables, and statistical analysis. The use of the 2017-18 BDHS data is appropriate for a nationally representative study. The criteria for participant selection are clearly outlined, and the inclusion and exclusion process is well-defined. The statistical methods, including Kaplan-Meier survival curves and modified Poisson regression, are appropriate for analyzing time-to-event data and handling the complex survey structure.  **Strengths of the Methodology:**  1. **Transparent Selection Criteria:** The criteria for selecting study participants are clear, contributing to the study's internal validity.  2. **Appropriate Statistical Methods:** The use of Kaplan-Meier survival curves and Poisson regression for analyzing time-to-event data is suitable for the research objectives.  **Critique of the Methodology:**   1. **Multicollinearity Check:** While the methodology mentions a multicollinearity check using Variance Inflation Factors (VIF), it would be helpful to report the results of this check or mention that no significant multicollinearity was found.   2. **Clarity on Duration:** The description of the "duration" in the time-to-event analysis could be further clarified for readers unfamiliar with this statistical approach.  Overall, the manuscript provides a comprehensive overview of the study, and the introduction and methodology are well-structured. Below are some feedback and suggestions on the Results and Discussion sections: | We have now reported relevant results in the supplementary file. Please checked the newly added supplementary tables.  We have added a definition of duration, please see-  *Duration of PPFP was defined as the month in which modern contraception was first initiated after the live birth.* |
| ### Results:  1. **Clarity in Presentation:**  - The background characteristics are well-presented in Table 1. However, consider breaking it down into sub-sections to improve readability. For example, you could have sub-sections for demographic characteristics and maternal healthcare utilization.  2. **Figures and Tables:**  - Figures 2, 3, 4, and 5 are referred to but not provided in the document. Make sure to include these figures for a complete understanding of the results.  - Ensure that the captions for figures and tables are sufficiently detailed to make them self-explanatory.  3. **Survival Probability Analysis:**  - The interpretation of the survival probability results could be made clearer. Explain the implications of the probabilities at different time points more explicitly. | We have now added sub-headings as per your suggestions to address your comment.  The figures you mentioned are already presented in the manuscript. Please see pages 16-18 to find them.  We have revisited captions and titles. They are self-explanatory now.  As the PPFP uptake is not significantly varied by place of residence and wealth quintile. We did not provide any details of those survival curves (figure 3 and 4). However, additional line is incorporated for the description of figure 5, please see the line-  *However, we found a variation in modern FP methods used in the postpartum period according to women’s level of education, with women with higher education levels more likely to use modern FP methods in the postpartum period than those with no education* |
| ### Discussion:  1. **Structure:**  - Consider breaking down the discussion into sub-sections to address specific aspects, such as challenges in maternal healthcare services, individual-level factors, and policy implications. This will enhance the organization and readability of the discussion.  2. **Linking to Literature:**  - Strengthen the discussion by linking your findings to existing literature more explicitly. If your results align or contrast with previous studies, discuss these points in detail.  3. **Policy Implications:**  - While you discuss policy implications, consider providing more actionable recommendations. How can policymakers address the identified challenges, and what specific changes could be made based on your findings?  4. **Addressing Limitations:**  - You've appropriately discussed the limitations of your study. It might be useful to discuss potential avenues for future research that could address these limitations.  5. **Causality and Temporality:**  - Acknowledge the cross-sectional nature of your study and the limitations it imposes on establishing causality. Discuss the need for prospective studies to confirm the observed associations.  6. **Language and Clarity:**  - Some sentences are complex and might benefit from simplification. Ensure that each point is expressed clearly and concisely.  7. **Conclusion:**  - The conclusion could be more concise and reiterate the key findings and their policy implications.  Remember that these are suggestions, and you should adapt them according to the specific guidelines of the journal you are submitting to. Overall, your manuscript is well-structured and addresses an important public health issue. Good luck with your submission! | As per the PLoS ONE guidelines, we are not allowed to do it. However, we have now separated several paragraphs to address this. Many thanks for your useful comment.  We have again revisited the discussion section and updated the citations to address your comment.  We have edited the relevant sentences to address your comment. They now read-  This would put Bangladesh in the challenge of not achieving the SDGs targets related to improving maternal and child health. Enhancing maternal healthcare services, integrating PPFP as a component of services offered by private healthcare facilities, and addressing structural challenges are recommended to improve the uptake PPFP. This needs more focus on healthcare services delivery system.  We have added the following sentences at the end of the strengths and limitation section to address your comment-  *Future research should be conducted by implementing a prospective study design along with a broad range of factors.*  We have already such recommendation to address your point 4.  We have thoroughly checked the entire manuscript again and made necessary edits to improve readability and clarity.  We have revisited the conclusion section and ensured that only the most important findings are presented there.  Many thanks to minutely read our paper. |
